# Indirect action to cell killing by SOBP carbon-ion beams

**DOI:** 10.1093/jrr/rrt192

**Published:** 2014-03

**Authors:** Ryoichi Hirayama, Yoshitaka Matsumoto, Akiko Uzawa, Yumiko Kaneko, Kana Koda, Masakuni Ozaki, Kei Yamashita, Huizi Li, Miho Noguchi, Toshiyuki Shirai, Yoshiya Furusawa

**Affiliations:** 1 Research Center for Charged Particle Therapy, National Institute of Radiological Sciences (NIRS), 4-9-1 Anagawa, Inage-ku, Chiba-shi, Chiba 263-8555, Japan; 2 International Open Laboratory, National Institute of Radiological Sciences (NIRS), Japan; 3 Advanced Science Research Center, Japan Atomic Energy Agency (JAEA), Japan

**Keywords:** SOBP carbon-ions, OH radical, indirect action, cellular lethality, RBE

## Abstract

Purpose: The aim of this study was to clarify the cell survival in human salivary gland (HSG) cells under oxic condition after 290 MeV/nucleon carbon-ion beams and 200 kV X-rays. Moreover, we examined OH radical-mediated indirect actions from either SOBP carbon beams or photon beams on cellular lethality.

Materials and methods: *Cell culture*: The HSG cells were grown in E-MEM (SIGMA) supplemented with 10% FBS and antibiotics under a humidified air with 5% CO_2_ at 37˚C. The cells were seeded into T25 cm^2^ flask (CORNING) at a concentration of 4 × 10^5^ cells per flask for 48 h prior to irradiation. *Irradiation and treatment with DMSO*: Carbon ions (^12^C^6+^) were accelerated by the HIMAC synchrotron to 290 MeV/nucleon. Depths in the SOBP beams were selected using a PMMA range shifter (116.10 and 143.84 mmH_2_O at middle and distal-end of SOBP, respectively). X-ray irradiations were performed using an X-ray generator (Shimadzu, Pantac HF-320S) operating at 200 kV and 20 mA, with a filter of 0.5 mm aluminum and 0.5 mm copper. The flasks were filled with 5 ml of medium containing different concentrations of DMSO ranging from 0 to 1.0 M for 1 h prior to irradiation. *Colony formation assay*: After irradiation, the cells were seeded into triplicate 60-mm plastic dishes at a density of ∼100 living cells per dish and incubated for 14 days. The colonies were fixed with 10% formalin solution, stained with 1% methylene blue solution and colonies consisting of more than 50 cells were counted. The 10% survival level (D_10_ or LD_90_) was calculated from a dose–response curve fitted by an LQ equation. *Calculation of the maximum protection by DMSO (DMSO method)*: The maximum degree of protection (DP), the concentration of DMSO that provides the maximum protection against cell killing, was calculated as well as our previous work [ 1– 4]. Briefly, the maximum DP was calculated by an extrapolation of reciprocals of surviving fractions over those of DMSO concentrations. The DP was defined by the below equation, and regression curves were drawn in the plots of DP as a function of DMSO concentration.

(1) $${\rm DP}=\displaystyle{{{\rm ln(SF}_{\rm 0} {\rm )} - {\rm ln(SF}_x {\rm )}} \over {{\rm ln(SF}_{\rm 0} {\rm )}}}, $$

where SF_0_ and SF_x_ are surviving fractions at 0 and *x* M of DMSO concentrations, respectively. The DP is expressed as the increase in the surviving fraction in the presence of DMSO normalized by the surviving fraction in the absence of DMSO (Fig. 1). Regression lines were drawn in the graphs of the reciprocals of DP plotted against those of DMSO concentration. The maximum DP is the value at the point of intersection of the regression line at the infinite concentration of DMSO.

Results: Colony forming assays were used to determine the surviving fractions of exponentially growing HSG cells at various doses of X-rays. The *D*_10_ value was 4.7. The *D*_10_ values for SOBP beam were 3.1 and 1.9 at middle and distal-end positions, respectively. The RBEs were 1.5 at middle and 2.5 at distal-end of SOBP. The contributions of indirect action to cell killing were 77% for X-rays, 80% at middle and 65% at distal-end of SOBP beam.

**Fig. 1.**
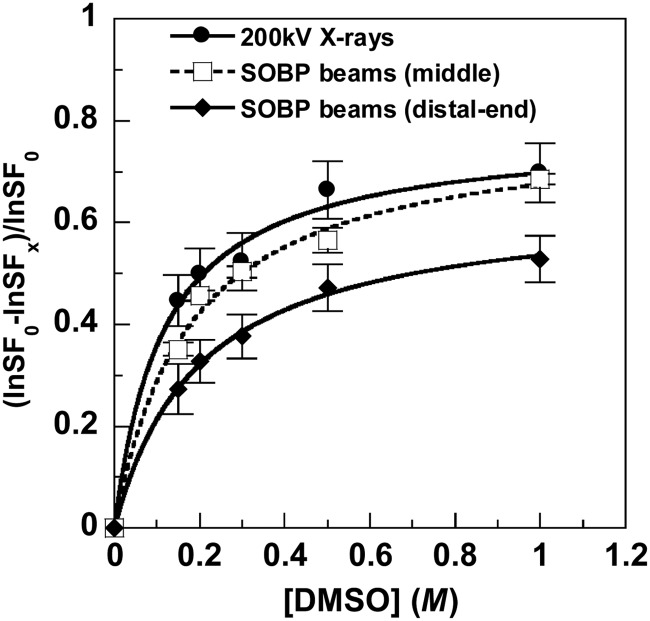
Effects of DMSO on the survival of HSG cells after exposure to X-rays or SOBP carbon beams. DPs were determined using Equation (1). The curves were fitted by the Michaelis–Menten kinetics. The error bars represent the standard errors.

Effects of DMSO on the survival of HSG cells after exposure to X-rays or SOBP carbon beams. DPs were determined using Equation (1). The curves were fitted by the Michaelis–Menten kinetics. The error bars represent the standard errors.

Summary: In this study, we could see the high contribution of indirect action to cell killing at the distal-end in SOBP carbon-ions, although RBE of 2.5 was shown.

Clinical Trial Registration number if required. No.

## References

[1] Ito A, Nakano H, Kusano Y (2006). Contribution of indirect action to radiation-induced mammalian cell inactivation: dependence on photon energy and heavy-ion LET. Radiat Res.

[2] Hirayama R, Ito A, Tomita M (2009). Contributions of direct and indirect actions in cell killing by high-LET radiations. Radiat Res.

[3] Hirayama R, Matsumoto Y, Kase Y (2009). Radioprotection by DMSO in nitrogen saturated mammalian cells exposed to helium ion beams. Radiat Phys Chem.

[4] Hirayama R, Ito A, Noguchi M (2013). OH radicals from the indirect actions of X-rays induce cell lethality and mediate the majority of the oxygen enhancement effect. Radiat Res.

